# Impact of Altered Gastrocnemius Morphometrics and Fascicle Behavior on Walking Patterns in Children With Spastic Cerebral Palsy

**DOI:** 10.3389/fphys.2020.518134

**Published:** 2020-10-07

**Authors:** Matthias Hösl, Annika Kruse, Markus Tilp, Martin Svehlik, Harald Böhm, Antonia Zehentbauer, Adamantios Arampatzis

**Affiliations:** ^1^Gait and Motion Analysis Laboratory, Schön Klinik Vogtareuth, Vogtareuth, Germany; ^2^Department of Biomechanics, Movement and Training Sciences, Institute of Human Movement Science, Sport and Health, University of Graz, Graz, Austria; ^3^Paediatric Orthopaedics Unit, Department of Orthopaedics and Trauma, Medical University of Graz, Graz, Austria; ^4^Gait Laboratory, Orthopedic Children’s Hospital Aschau, Aschau im Chiemgau, Germany; ^5^Human Movement Science, Faculty of Sports Science, Ruhr University Bochum, Bochum, Germany; ^6^Department of Training and Movement Sciences, Humboldt University of Berlin, Berlin, Germany; ^7^Berlin School of Movement Science, Humboldt University of Berlin, Berlin, Germany

**Keywords:** cerebral palsy, ultrasonography, triceps surae, muscle architecture, paresis, toe-walking, crouch gait

## Abstract

Spastic cerebral palsy (SCP) affects neural control, deteriorates muscle morphometrics, and may progressively impair functional walking ability. Upon passive testing, gastrocnemius medialis (GM) muscle bellies or fascicles are typically shorter, thinner, and less extensible. Relationships between muscle and gait parameters might help to understand gait pathology and pathogenesis of spastic muscles. The current aim was to link resting and dynamic GM morphometrics and contractile fascicle behavior (both excursion and velocity) during walking to determinants of gait. We explored the associations between gait variables and ultrasonography of the GM muscle belly captured during rest and during gait in children with SCP [*n* = 15, gross motor function classification system (GMFCS) levels I and II, age: 7–16 years] and age-matched healthy peers (*n* = 17). The SCP children’s plantar flexors were 27% weaker. They walked 12% slower with more knee flexion produced 42% less peak ankle push-off power (all *p* < 0.05) and 7/15 landed on their forefoot. During the stance phase, fascicles in SCP on average operated on 9% shorter length (normalized to rest length) and displayed less and slower fascicle shortening (37 and 30.6%, respectively) during push-off (all *p* ≤ 0.024). Correlation analyses in SCP patients revealed that (1) longer-resting fascicles and thicker muscle bellies are positively correlated with walking speed and negatively to knee flexion (*r* = 0.60–0.69, *p* < 0.0127) but not to better ankle kinematics; (2) reduced muscle strength was associated with the extent of eccentric fascicle excursion (*r* = −0.57, *p* = 0.015); and (3) a shorter operating length of the fascicles was correlated with push-off power (*r* = −0.58, *p* = 0.013). Only in controls, a correlation (*r* = 0.61, *p* = 0.0054) between slower fascicle shortening velocity and push-off power was found. Our results indicate that a thicker gastrocnemius muscle belly and longer gastrocnemius muscle fascicles may be reasonable morphometric properties that should be targeted in interventions for individuals with SCP, since GM muscle atrophy may be related to decreases in walking speed and undesired knee flexion during gait. Furthermore, children with SCP and weaker gastrocnemius muscle may be more susceptible to chronic eccentric muscle overloading. The relationship between shorter operating length of the fascicles and push-off power may further support the idea of a compensation mechanism for the longer sarcomeres found in children with SCP. Nevertheless, more studies are needed to support our explorative findings.

## Introduction

Spastic cerebral palsy (SCP) is a neuromuscular disorder due to a nonprogressive brain lesion occurring early in infancy or before (Graham et al., 2016). As a result, patients with SCP often present hypertonia, hyperreflexia, and impaired motor control (Lee et al., 2014). Alterations of the musculoskeletal system, e.g., muscle weakness, restricted joint range of motion (RoM), and increased passive joint stiffness, are partly attributable to altered muscle-tendon properties (Barber et al., 2012). This likely contributes to limited mobility and restricted participation in daily life (Damiano et al., 2000).

Main intervention goals in the therapy of individuals with SCP are to reduce spasticity and to counteract the development and deterioration of muscular pathology. Thus, the plantar flexor muscle-tendon complex is frequently targeted during treatment, e.g., by physical therapy, surgeries, serial casting, orthoses, or botulinum toxin injections. In comparison to typically developing (TD) peers, plantar flexor muscles are macroscopically smaller and shorter in patients with SCP (Malaiya et al., 2007; Barber et al., 2011; Noble et al., 2014). For the gastrocnemius medialis (GM), reductions in muscle volume (Barber et al., 2013; Herskind et al., 2016), fascicle length (Barber et al., 2013; Hösl et al., 2015; Frisk et al., 2019), and physiological cross-sectional area (Barber et al., 2013; Herskind et al., 2016) have been frequently documented. In addition, tendon properties are changed, e.g., increased length (Wren et al., 2010; Hösl et al., 2015; Kruse et al., 2018) and reduced cross-sectional area (Gao et al., 2011; Kruse et al., 2018), have been reported. Examinations on microscopic level demonstrated longer sarcomeres (Lieber and Friden, 2002; Ponten et al., 2007; Smith et al., 2011; Mathewson et al., 2014; Mathewson and Lieber, 2015) and reduced serial sarcomere number (Lieber and Friden, 2018). These alterations likely limit muscle force output and excursion, which may in turn deteriorate walking.

Patients with SCP often walk slow, with increased energy demands (Kerr et al., 2008) and constrained or excessive joint movements with different gait characteristics (Armand et al., 2016; Zhou et al., 2017). Although the natural, untreated progression of gait pathology generally remains difficult to track in children with SCP, with higher age, crouch gait, a gait pattern with excessively flexed knees, might often increase (Bell et al., 2002; Gough et al., 2004; Rose et al., 2010). Crouch gait affects 45–60% of independently ambulant patients (Rethlefsen et al., 2017) with higher rates (74–88%) found in bilaterally affected patients (Wren et al., 2005), while equinus gait, characterized by ground contact with the forefoot or midfoot and subsequent lack of dorsiflexion, is present in nearly every second ambulant child with SCP. Concerning the force output during gait, the plantar flexors usually supply the major forward drive in healthy subjects, but during gait of patients with SCP, marked reductions in ankle joint power during push-off is a typical issue (Dallmeijer et al., 2011; Eek et al., 2011).

To date, there is yet a lack of information about the specific muscle properties in SCP that could be decisive for particular gait deviations. During gait, the plantar flexors generally control tibia progression, generate propulsion, and accelerate the forward swinging leg (Neptune et al., 2001; Arnold et al., 2005; Santuz et al., 2017). Distinguishing the gastrocnemius and soleus, both seem to similarly contribute to vertical support during unimpaired gait, but during the late stance phase the gastrocnemius induces forward acceleration while the soleus contributes to braking of forward velocity throughout mid‐ and terminal stance (Francis et al., 2013). Most notably, the gastrocnemius is a bi-articular muscle with the potential capacity to generate knee flexion upon stimulation, too (Lenhart et al., 2014). Overall, in SCP, a thinner GM muscle or triceps surae seems to be related to reduced gross motor function (Ohata et al., 2006; Choe et al., 2018). Moreover, the gastrocnemius muscle volume is smaller in children without independent walking skills (Herskind et al., 2016). In addition, plantar flexors of children with SCP show decreased concentric (Ross and Engsberg, 2002) or isometric strength (Wiley and Damiano, 1998; Elder et al., 2003; Stackhouse et al., 2005; Downing et al., 2009) during instrumented strength tests. Still, more detailed knowledge about the actual relationship of muscle structure to biomechanics of gait is needed. Fairly recently, Martin Lorenzo et al. (2018) captured passive gastrocnemius fascicle length with ultrasound aiming to explain the amount of reduced propulsive ankle joint power during gait (Martin Lorenzo et al., 2018). However, they could not find statistically significant associations. Frisk et al. (2019) speculated that reduced fascicle lengths are essential contributors to reduced torque generation in the gastrocnemius in SCP. They assumed that a more plantar-flexed position during gait (~more equinus) would take advantage of the underlying alteration of the torque-angle relation (Barber et al., 2012). Thus, children with shorter fascicles may opt to walk in larger plantar flexion to avoid unfavorable length-force relationships of their sarcomeres. However, the understanding may be compromised by a lack of knowledge about the muscle fascicles’ behavior during gait, which they did not simultaneously examine.

Ultrasound studies distinguished the muscle and tendinous behavior *in vivo*. They demonstrated that, in healthy heel-toe gait, the GM or soleus fascicles (Fukunaga et al., 2001; Ishikawa et al., 2005; Aggeloussis et al., 2010; Rubenson et al., 2012; Barber et al., 2017) shorten during loading response, the gait cycle’s first period of double-limb support after initial ground contact. Thereafter, they display a subsequent active stretch-shorten cycle with near isometric or minor eccentric length change during single stance and shortening during push-off. During this cycle, the tendon stretches to a larger extent and stores elastic strain energy that is released during recoil. Since the tendon undergoes most of the whole muscle-tendon unit (MTU) length change, the muscles are able to contract at favorable lengths (at or near the plateau region of their force-length curve) and at a slower speed for force production (Fukunaga et al., 2001). The tendon stiffness appears crucial for the efficiency of this interaction (Lichtwark and Wilson, 2008).

Knowledge about the contractile mode of the spastic plantar flexors during gait has been often theory-driven, based on expertise (Gough and Shortland, 2012), forward simulations (Neptune et al., 2007), or musculoskeletal modeling (van der Krogt et al., 2015; Rha et al., 2016). While structural shortening of the spastic plantar flexor MTU derived from musculoskeletal modeling has been primarily linked with equinus gait (Eames et al., 1997), among other factors (Delp et al., 1996; Hicks et al., 2008), a shorter and passively less extensible gastrocnemius MTU is reflected by increased crouch gait overtime (Maas et al., 2015).

Fairly recently, ultrasonography was also used to investigate the *in vivo* walking behavior of the GM muscle in children with SCP (Hösl et al., 2016; Kalsi et al., 2016; Barber et al., 2017). However, the studies reported seemingly inconsistent results. Kalsi et al. (2016) found increased lengthening of the gastrocnemius muscle belly during single support in comparison to their TD peers. Barber et al. (2017) delivered evidence of increased fascicle lengthening during the early and mid-stance phase; however, contrastingly, Hösl et al. (2016) did not find increased lengthening excursions but less fascicle shortening during push-off in accordance with Barber (2017). The inability to resist tensile forces was assumed to result in energy absorbed by the muscle rather than stored by the tendon, potentially damaging the muscle on the long term, which was in line with earlier speculations about harmed fiber growth (Gough and Shortland, 2012) or promoted fibrosis (Pitcher et al., 2015) due to eccentric overloading in SCP. In this context, three aspects warrant further investigation: a different strength level may potentially be related to the extent of eccentric fascicle lengthening. Additionally, in the studies of Kalsi et al. (2016) and Barber et al. (2017), children also primarily presented alterations in the ankle joint (equinus gait), while in Hösl et al. (2016) patients walked on average with increased knee flexion. Since a steeper landing angle of the foot (i.e., more toe walking) may generally exert larger tensile forces on the gastrocnemius muscle-tendon complex, the walking pattern might play a crucial role. Moreover, healthy populations show a speed-dependent modulation of the contractile modes (Ishikawa et al., 2005), e.g., greater lengthening of the GM muscle belly during running than during walking (Ishikawa et al., 2005).

Evidence on the contractile fascicle behavior during gait and its relation to ankle kinetics is scarce. Barber et al. (2017) hypothesized that since muscle fascicles in SCP shorten less during late stance, they induce less ankle joint power. Also, Frisk et al. (2019) found less fascicle shortening during supramaximal stimulations simultaneously with reduced torque generation in SCP patients, but they were questioning if this would affect muscle force output during gait. Next to that, the implications of shorter relative operating length (Hösl et al., 2016) were not investigated. In TD individuals, the gastrocnemius fascicles usually operate at shorter relative length at faster walking (Ishikawa et al., 2005). This shift toward the ascending limb of the force-length relationship decreases muscle force output but may support the storage of elastic energy (Ishikawa et al., 2005) to augment the power output at the ankle joint during faster walking (Schwartz et al., 2008). Based on findings of longer sarcomeres and reduced sarcomeres in series in SCP (Lieber and Friden, 2002; Ponten et al., 2007; Smith et al., 2011; Mathewson et al., 2014; Mathewson and Lieber, 2015), the sarcomeres in SCP may likely need to operate with less overlap. Maintaining shorter fascicle operating length could thus be vital to produce push-off. Furthermore, fascicle shortening velocities have not yet been examined in SCP muscles. However, Farris and Sawicki (2012) stated that GM fascicle shortening velocity during stance may be a key factor for the speed healthy humans choose to walk at. Biomechanically, higher fascicle velocities during stance phase could actually impair the fascicles’ ability to produce force.

In this study, we wanted to link resting GM muscle morphometrics and the contractile fascicle behavior (both in length and velocity) during walking in children with SCP to kinematic and kinetic determinants of gait and muscle function. Due to the neuromuscular impairment in children with SCP, we were also keen to see whether the relationships were the same as in healthy controls. We performed a further analysis of the sample presented in Hösl et al. (2016). Data of children with SCP and their TD peers captured during both rest and during gait were analyzed. Ankle joint kinetics were already presented by Barber et al. (2017); however, the associations of the fascicle contractile modes and gait kinetics were not established. We thus complemented the ultrasound-based contractile behavior of the gastrocnemius fascicles during gait by Hösl et al. (2016) with ankle joint kinetics from inverse dynamics during overground gait of the same participants. Three aims were deduced: first, we aimed to study the relationship between alterations in resting-muscle fascicle length as well as muscle thickness (MT) and joint excursion as well as muscle function, e.g., during push-off. We assumed that children with SCP with more severe muscle pathology, namely, shorter resting GM muscle fascicle lengths and thinner gastrocnemius muscle bellies, will walk slower and less erected (with more knee flexion) and provide less push-off power. With reference to Frisk et al. (2019), we also expected that children with shorter fascicles will walk in more plantar flexion. Second, we aimed to further explore the divergent findings on eccentric fascicle lengthening in SCP during gait. We expected larger fascicle lengthening in weaker children with SCP, in those with a steeper foot landing angle (i.e., an index for more severe forefoot landing), and in slower walkers. The third aim of our study was to investigate if the fascicle dynamics, i.e., operating length, shortening excursion, or velocity, are related to reductions in ankle push-off power. We expected that a longer operating length, less fascicle shortening excursion, and faster shortening velocities will be related to smaller push-off power in the SCP and TD children. Eventually, due to its exploratory nature, this study aims to generate further research goals to be studied.

## Materials and Methods

In the following, a brief description of the participant characteristics and study protocol can be found. For detailed information, we refer to Hösl et al. (2016).

### Participants

Fifteen children with bilateral SCP (mean age: 11 ± 2.8 years, four females) and 17 TD controls (mean age: 12.2 ± 2.3 years, eight females) participated in the study. Eleven children with SCP were classified as GMFCS level I and four children as GMFCS II. Exclusion criteria were any leg surgeries or botulinum toxin injections within the last 12 months. Only data of the more involved side (i.e., less passive dorsiflexion) were included. Further anthropometric details can be found in Hösl et al. (2016). For the TD controls, the right side was chosen. Experiments received ethical approval by the Technical University Munich, Germany, and informed written consent was obtained (Hösl et al., 2016).

### Protocol

The participants were physically examined (for passive RoM and modified Ashworth scale [Bohannon & Smith, 1987] at the knee and ankle) and performed isometric strength tests and a 3D gait analysis on a treadmill, as well as during overground walking. During the physical exam, passive dorsiflexion was measured using ruler-based goniometry. Moreover, a seated rest measurement was performed with the knees 90° flexed and ankles in neutral alignment (foot flat on the ground) to assess the GM muscle morphometrics at rest and extract the resting length of fascicles. Subjects were encouraged to relax and based on their feedback that they did not feel any tension. Although influenced by hypertonia and contracture, the modified Ashworth scale was used as a surrogate measure of spasticity. Since we did the resting measurement once with electromyography (EMG) and once with ultrasound (Hösl et al., 2016), we can also confirm that the muscle was silent. Peak isometric plantar flexor force (N/kg) was assessed by use of a handheld dynamometer and a uniaxial force sensor (Mobi, Sakaimed, Tokyo, Japan) during five maximum voluntary contractions (MVC, 3-s contraction, 1 min rest). For these, participants were long-seated (hips semi-flexed, knees extended, and ankle as close as possible to neutral). After discarding the lowest and highest values, three trials were averaged. No motion capture data were collected during the strength tests. For the current analysis, data captured during the flat-forward walking on both the treadmill and overground were included (Hösl et al., 2016). Since the treadmill was un-instrumented, the gait kinetics were extracted from the overground walking trials.

### Gait Analysis

In both conditions, gait analyses were performed at the children’s preferred walking speed. A Nexus system (Vicon Inc., Oxford, UK) with eight MX-Cameras was used to capture lower-limb kinematics using a modified plug-in gait model (Stief et al., 2013). Marker data were sampled at 200 Hz. On the treadmill (Atlantis, Heinz Kettler, Ense-Parsit, Germany), the participants wore a harness (h/p/cosmos, Nussdorf-Traunstein, Germany) without weight support, which was connected to a safety frame (Mobil Konzept Loadmaster 80, RehaMed Technology, Dietzenbach, Germany). After a familiarization period, data were captured during 10 s of treadmill walking. During the overground condition, the children walked up and down a 12-m-long flat walkway and ground reaction force data were captured at 1000 Hz *via* two force plates (AMTI, Watertown, United States). Three or more consistent kinetic trials were averaged.

### Ultrasound and Electromyography

**Figure 1:**
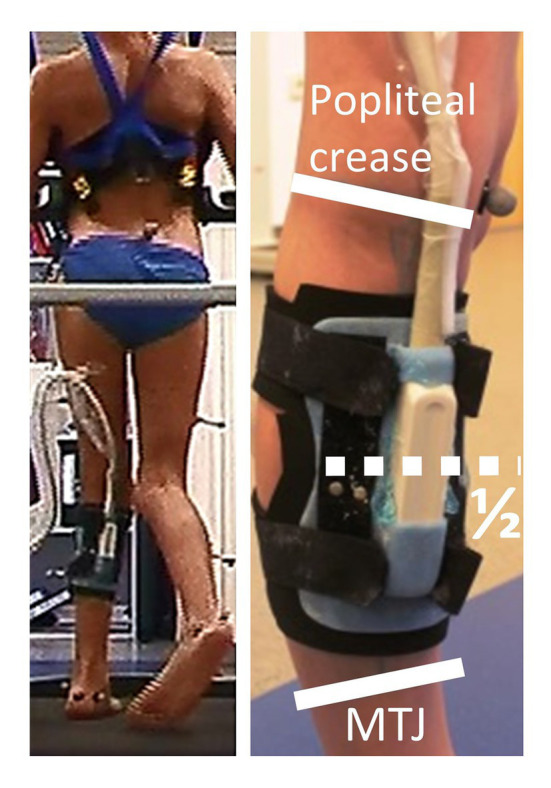
Example of a child with SCP during treadmill gait and the ultrasound probe fixated at the calf and markers from 3D motion capturing. The safety harness (h/p/cosmos, Germany) did not provide bodyweight support. The ultrasound probe Echoblaster 128 ultrasound (Telemed, Vilnius, Lithuania) was attached with a custom-made plastic cast, covered with neoprene, and firmly attached to the shank with Velcro straps, halfway between popliteal crease and the gastrocnemius muscle-tendon junction (MTJ).

An Echoblaster 128 ultrasound (Telemed, Vilnius, Lithuania) was used to image the GM muscle fascicles at 60 Hz with a linear probe (8 MHz, field of view of 60 mm) during gait (Figure 1). The probe was held in place with a plastic cast covered with neoprene. Measurements of the fascicle lengths were made at a mid-belly position (i.e., half-way between muscle-tendon-junction and popliteal crease), and the scanner was aligned according to Benard et al. (2009). The ultrasound was synchronized with the motion capture data *via* a pulse that was fed to a DTS EMG System (Noraxon, Scottsdale, United States). Surface electrodes (Blue Sensor N, Ambu, Ballerup, Denmark) were placed on the muscle bellies of the GM, soleus, and tibialis anterior, and signals were sampled at 1000 Hz. EMG and ultrasound were captured in separate trials. A sequence of six successive strides was cropped into separate gait cycles. Ultrasound videos of each gait cycle were then analyzed separately. Fascicle lengths were measured with a tracking algorithm (Gillett et al., 2013), and subsequent frame-wise inspection by the same investigators (AZ and MH) and corrections were manually made were necessary. Gait data were interpolated to 100 points across each stride, and an average for each participant and condition was determined.

Furthermore, resting-muscle measurements were performed during seated rest defined with the knees in 90° flexion and the ankle in a neutral position (foot flat on the ground). In addition, the muscle-belly thickness was measured in the same resting position. During gait, all morphometric variables were normalized to the resting lengths and all resting values were normalized to shank length. Further information about the post-processing e.g., calculation of MTU length (Orendurff et al., 2002) and EMG processing (Panizzolo et al., 2013) can be found elsewhere (Hösl et al., 2016). However, the information about GM MTU length and EMG activity was included for illustrative purposes in the present study.

### Outcomes

Self-selected walking (SSW) speed was presented as absolute values (m/s). For kinematics, we extracted the foot landing angle (i.e., foot to floor landing angle at initial contact), the mean dorsiflexion, and knee extension during stance. Concerning joint kinetics during gait, joint moments were normalized to body mass (Nm/kg). We further extracted the positive peak ankle joint power (W/kg) during push-off. For the gastrocnemius morphometries, we selected the resting muscle belly thickness and fascicle length during seated rest. For quantifying the working mechanisms during gait, we calculated the mean fascicle operating length (% resting length) in stance, as well as the extent of eccentric excursion during single stance and the concentric excursion during push-off. Furthermore, the respective shortening velocity was calculated during push-off (expressed in % resting length/s). We further computed the mean isometric plantar flexor strength (N/kg) from three trials as described above.

### Statistics

Participant characteristics, physical exam results, and muscle morphometrics during rest and gait were compared between children with SCP and TD children by use of unpaired *t*-tests or Mann-Whitney U tests if the variables were not normally distributed. With regard to the associations, we correlated the resting muscle belly thickness and fascicle lengths with the SSW, the kinematics, and the peak push-off power. For eccentric lengthening, we tested the correlations to isometric plantar flexor strength, the landing angle of foot, and the SSW. For dynamic fascicle behavior, we tested the associations of relative fascicle operating length, fascicle shortening excursion, and fascicle shortening speed to the peak power during push-off. All bivariate relationships were tested both for SCP and for TD children by use of Pearson correlations or, if not normally distributed, by Spearman-rank correlations. Correlation coefficients were interpreted as poor (<0.2), fair (0.21–0.4), moderate (0.41–0.6), and good (0.61–0.8) following the guidelines by Altman (1990). Normal distribution was tested with Shapiro-Wilk tests. The alpha level was set to 0.05 for group comparisons and during correlations corrected for familywise comparisons according to Šidák (1967) as follows: *α*_sid_ = 1 − (1 − 0.05)^1/K^, with *K* being the number of dependent variables for each set of correlations. Thus, the Šidák-adjusted level (*α*_sid_) was 0.0127 for correlations with resting morphometrics and 0.0170 for all other sets of tests. If directed hypotheses were formulated, one-tailed tests were performed.

## Results

The findings are displayed as means ± standard deviations. Compared to their TD peers, children with SCP displayed significant reductions in passive dorsiflexion when measured with extended knees during the clinical exam (1 ± 8° and 15 ± 4° in SCP and TD, respectively, *p* < 0.01) accompanied by a modified Ashworth scale score of 2 ± 0.8 (range 1–3).

**Figure 2:**
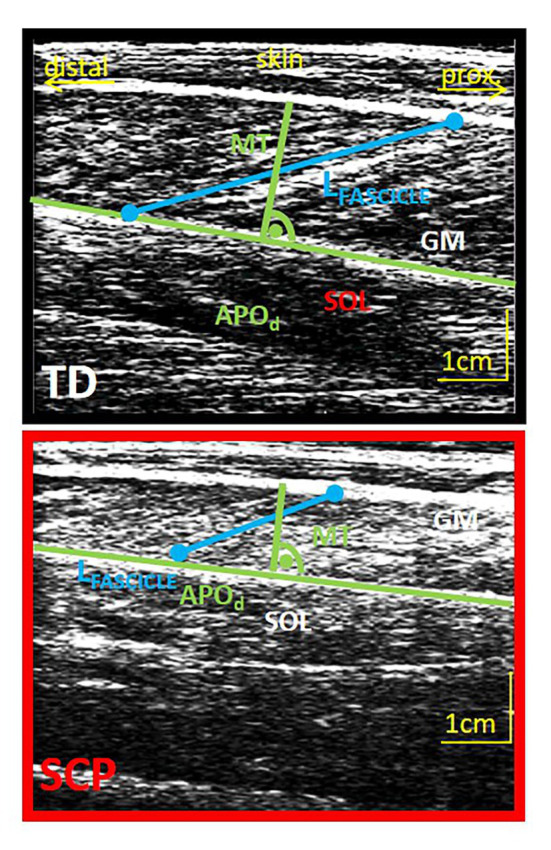
Example of an ultrasound image of the GM muscle belly of a TD child (12 years, upper image) and a child with SCP (13 years, lower image) and schematic extraction of parameters of interest. Green: determination of muscle thickness (MT) at the midbelly portion perpendicular to the deep aponeurosis. Blue, fascicle length; APO_d_, distal aponeurosis; GM, gastrocnemius medialis; SOL, soleus.

Their reference fascicle lengths and muscle-belly thickness, both measured during seated rest, were reduced by 18% (both *p* ≤ 0.016). An example of an ultrasound image for both groups is shown in Figure 2.

During overground walking, the self-selected speed was 1.15 ± 0.17 m/s in SCP and 1.31 ± 0.14 m/s in TD (*p* < 0.01). With respect to the overground walking, the decrease in comfortable speed on the treadmill was similar (*p* = 0.71) between the TD children and the children with SCP (5 ± 10% and 6 ± 10%, respectively).

**Figure 3:**
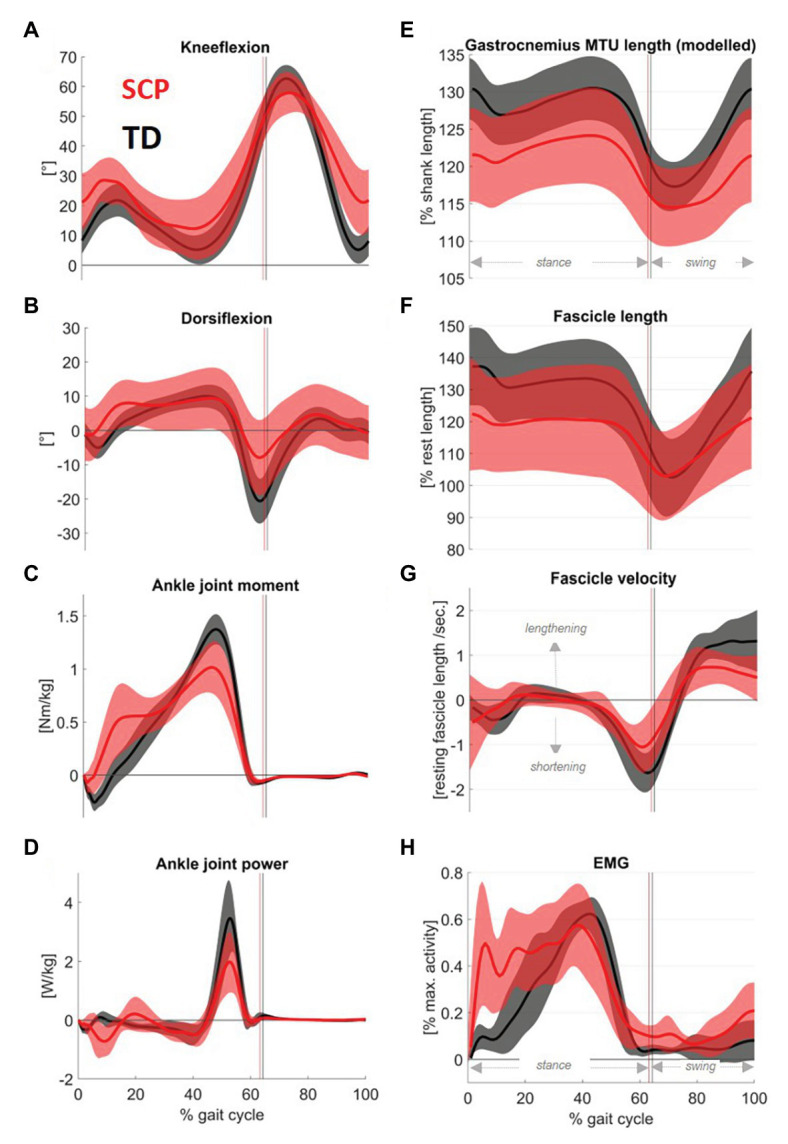
Mean traces (shaded area: 1SD) of sagittal plane **(A)** knee and **(B)** ankle angles, as well as **(C)** ankle joint moments and **(D)** ankle joint power in children with SCP (red) and their TD peers (black). The figure further displays **(F)** the gastrocnemius fascicle length changes throughout the gait cycle and **(G)** the calculated fascicle velocity. For the sake of completeness, the graphs were complemented by **(E)** modeled GM muscle-tendon unit (MTU) length and **(H)** the gastrocnemius activity assessed with electromyography. Electromyography (EMG) was rectified, filtered, and normalized on the max. Activity of all walking trials for each individual.

Children with SCP walked in a more crouched posture than their TD peers, characterized by significantly (*p* = 0.02) increased knee flexion (+6°) and slightly higher dorsiflexion (+1°) during stance (Figures 3A,B). The resulting kinematically modelled MTU length is shown in Figures 3E. In addition, seven out of 15 children with SCP landed with their forefoot first. Concerning the ankle moment (Figures 3C) and the ankle joint power (Figures 3D), the peaks during push-off were reduced by 20.5% and 42.0%, respectively (both *p* < 0.01).

**Figure 4:**
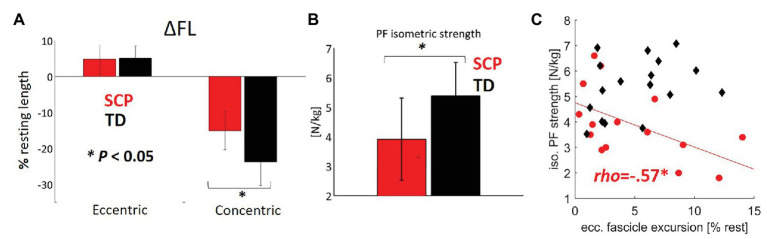
**(A)** Extent of eccentric and concentric fascicle excursions (ΔFL calculated with regard to the resting fascicle lengths) in children with SCP (red) and TD children (black). **(B)** Plantar flexor strength. **(C)** Relationship between the plantar flexor (PF) strength and eccentric fascicle excursion. ^*^p < 0.05 for group comparisons concerning ΔFL and isometric strength. ^*^p < 0.017 for correlations (with Šidák-adjusted level). NB, only regression lines for sign. Correlations are shown for each group.

The children with SCP reached 9% (*p* = 0.04) shorter maximum fascicle lengths (Figure 3F) and showed 37% less concentric fascicle shortening during push-off (Figure 4A, *p* = 0.001) with no significant group differences in eccentric fascicle excursion during stance (Figure 4A, *p* = 0.57).

Furthermore, the peak fascicle shortening velocity (Figure 3G) during push-off was 30% slower in the children with SCP (*p* < 0.01).

Figure 3H displays the simultaneous electromyographic muscular activity. Moreover, the isometric strength of the children with SCP was decreased by 27% (Figure 4B, *p* < 0.01). Resting fascicle lengths showed a significant positive correlation of good strength with SSW speed in children with SCP (Table 1, r = 0.61, *p* = 0.008). Furthermore, thicker muscle bellies were also significantly positively correlated with walking speed (Table 1, *r* = 0.60, *p* = 0.01).

**Table 1:** Correlation coefficients [Pearson’s *r* (italic font) and Spearman’s rho (upright font)] between gastrocnemius medialis (GM) muscle belly morphometrics at rest and gait-related outcomes in children with spastic cerebral palsy (SCP) and typically developing (TD) children.

Variables	Group	SSW (m/s)	DF (°)	KF (°)	PF power (W/kg)
Fascicle length (% shank length)	SCP	0.61^*^	*−0.55*	−0.66^*^	0.46
	TD	*0.02*	−0.03	*−0.38*	*0.03*
Muscle thickness (% shank length)	SCP	0.60^*^	*−0.57*	−0.69^*^	0.19
	TD	−0.12	0.11	−0.16	−0.16

* *p* < 0.0127 (with Šidák-adjusted level).

SSW, self-selected walking speed; DF, mean dorsiflexion during stance; KF, mean knee flexion during stance; PF power, peak ankle joint power. Background color defines the strength of correlation <0.2, poor (red); 0.21–0.4, fair (yellow); 0.41–0.6, moderate (light green); 0.61–0.8, good (dark green). Significant values are also displayed in bold.

**Figure 5:**
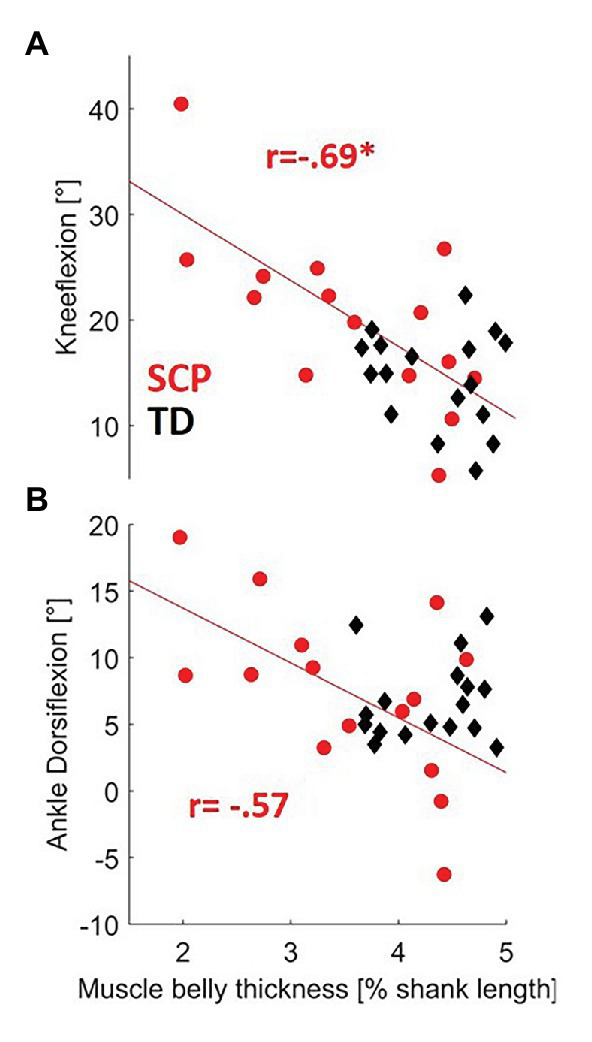
Relationship between resting-MT and both **(A)** knee flexion and **(B)** dorsiflexion during gait in children with SCP (red circles) and TD children (black diamonds). ^*^p < 0.0127 (with Šidák-adjusted level). NB, regression lines for MT for SCP failed to reach significance to dorsiflexion (*p* = 0.0284).

The moderate positive correlation between resting fascicle length and push-off power in children with SCP failed to reach statistical significance after Šidák correction (Table 1, *p* = 0.042). However, negative correlations of good statistical strength were found between longer fascicles as well as thicker muscle bellies and knee flexion (both *p* ≤ 0.004) in children with SCP. The negative correlations between fascicle length as well as MT with the dorsiflexion angle failed to reach significance. No significant correlation between the resting-muscle morphometrics and the gait parameters was found in the TD children (Table 1, Figure 5).

Despite no significant group difference in eccentric fascicle excursion in children with SCP and TD (Figure 4A), there was a moderate negative correlation between the amount of eccentric excursion and the plantar flexor strength (rho = −0.57, *p* = 0.015) only in the patients with SCP (Figure 4B, Table 2), indicating that weaker children with SCP experienced larger fascicle lengthening during the stance phase of gait. The landing angle of the foot or walking speed did not show a significant correlation with the extent of eccentric fascicle excursion (Table 2).

**Table 2:** Correlation coefficients [Pearson’s *r* (italic font) and Spearman’s rho (upright font)] between GM eccentric fascicle excursion during gait and both plantar flexor (PF) strength and gait-related outcomes in children with SCP and TD children.

Variables	Group	PF force (N/kg)	SSW (m/s)	Foot landing angle (°)
Eccentric excursion (% resting length)	SCP	−0.57^*^	−0.21	−0.08
	TD	*0.36*	*−0.35*	0.42

* *p* < 0.017 (with Šidák-adjusted level).

PF force, maximum isometric strength from handheld dynamometry; SSW, self-selected walking speed. Background color defines the strength of correlation <0.2, poor (red); 0.21–0.4, fair (yellow); 0.41–0.6, moderate (light green); 0.61–0.8, good (dark green). Significant values are also displayed in bold.

**Figure 6:**
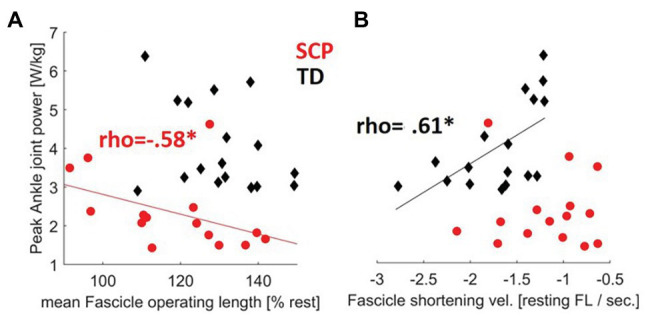
Contractile determinants of push-off power: relationships between the **(A)** fascicle operating length during gait and the **(B)** fascicle shortening velocity during gait with the propulsive ankle joint power in children with SCP (red circles) and TD children (black diamonds). ^*^p < 0.017 for correlations (with Šidák-adjusted level). NB, only regression lines for sign. Correlations are shown for each group.

Normalized operating length of the fascicles moderately negatively correlated with peak push-off power at the ankle joint in children with SCP (Table 3, Figure 6A, rho = −0.58, *p* = 0.013) indicating that the shorter the fascicles with respect to the resting length, the more push-off power may be generated. Furthermore, the fascicle shortening velocity or excursion did not show a significant correlation with the plantar flexion power during propulsion in the children with SCP (Table 3, Figure 6B), while in the TD children, a significant good association was found between slower shortening velocity of the fascicles and larger push-off power (Figure 6B, rho = 0.61, *p* = 0.0054). No significant correlation was found for SSW.

**Table 3:** Correlation coefficients [Spearman’s rho (upright font)] between GM fascicle dynamics during gait and ankle joint push-off power (PF power).

Variables	Group	Mean operating length (% resting length)	Concentric fascicle excursion (% resting length)	Max.shortening speed (resting length /s)
PF power (W/kg)	SCP	−0.58^*^	−0.01	-0.00
	TD	−0.23	−0.43	0.61^*^

* *p* < 0.017 (with Šidák-adjusted level).

Background color defines the strength of correlation <0.2, poor (red); 0.21–0.4, fair (yellow); 0.41–0.6, moderate (light green); 0.61–0.8, good (dark green).

## Discussion

In this study, we aimed to link resting GM muscle morphometrics and the contractile fascicle behavior (both excursion and velocity) during walking in children with SCP to kinematic and kinetic determinants of gait and muscle function. One main finding was that longer resting-muscle fascicles and thicker muscle bellies were positively correlated with walking speed. Furthermore, our findings suggest that longer resting fascicles and thicker muscle bellies may contribute to less flexed knees but may not normalize ankle kinematics. In addition, our results may suggest that reduced muscle strength seems to be an important factor related to an increase in eccentric excursion by the spastic paretic muscle during gait. Finally, our analyses indicated that an association between shorter operating length of the fascicles and more push-off power at the ankle may exist.

Focusing on resting-muscle morphometrics, children with SCP with longer gastrocnemius fascicle lengths and thicker muscle bellies walked faster and, as hypothesized, more erected (i.e., with less crouch gait). Concerning fascicle lengths, these findings are somewhat in line with biomechanical advantages of longer fascicles, extending their range of active force production (Blazevich and Sharp, 2005; O’Brien, 2016). Since at a given fascicle shortening velocity, the shortening of each sarcomere is less in longer fascicles, relative contractile velocity is lower and its force potential can be larger (Blazevich and Sharp, 2005). In computer simulations, next to other muscles that mainly contribute to an erected gait, e.g., the soleus or the glutei (Hicks et al., 2008; Ong et al., 2019), also the gastrocnemius vertically accelerates the center of mass, thus contributing to a more upright gait (Steele et al., 2013). Moreover, additional sarcomeres in series may also increase the passive muscle extensibility (Butterfield, 2010). Longer gastrocnemius fascicles could thereby allow children with SCP to walk more erected. Whether longer fascicles contain more sarcomeres remains debatable in SCP; however, benefits of longer passive fascicle length have been reported previously in SCP: longer rectus femoris muscle fascicles were related to higher sports‐ and physical functioning scales (Moreau et al., 2010), whereby longer fascicle lengths in the tibialis anterior were significantly related to faster walking (Bland et al., 2011). Our finding therefore supports the importance of longer fascicles also for the function of the GM in individuals with SCP. However, in contrast to our expectation and therefore also in contradiction of the speculations by Frisk et al. (2019), we did not find a positive correlation between longer fascicle lengths and more dorsiflexion in children with SCP. Our hypothesis was therefore rejected.

More deteriorated muscular pathology of the gastrocnemius as indicated by a thinner muscle belly and shorter fascicles was associated with a more pronounced knee flexion. This may usually be noted as a sign of progressive functional decline in individuals with SCP (Bell et al., 2002). Musculoskeletal modeling showed that the MTU length of the gastrocnemius can be short in flexed knee gait (Rha et al., 2016) or in equinus gait (Wren et al., 2004). Both often lead to recession surgeries in SCP. Although treatments aim to restore dorsiflexion and not to normalize muscle-tendon architecture (Sees and Miller, 2013), it needs to be taken into account that gastrocnemius’ fascicles or muscle belly thickness may undergo considerable atrophy due to an intervention as shown after Botulinum toxin application (Park et al., 2014), surgeries (Shortland et al., 2004), and splinting (Hösl et al., 2015). The associations found in this study may suggest that inducing gastrocnemius muscle atrophy due to treatment could help to correct excessive plantar flexion in children with bilateral SCP but may also cause a risk to induce undesired knee flexion during gait. With all due reservations considering the cross-sectional nature of this study, whether children show progressive crouch gait pathology could thus be influenced by preservation of muscle size.

Concerning the association between the gastrocnemius MT or the muscle fascicle lengths and peak ankle joint power, we could find a significant relationship in neither the individuals with SCP nor their TD peers. However, peak ankle power was reduced by −42% in the SCP group. Previous studies in healthy individuals showed that the active isometric plantar flexor force is proportional to their cross-sectional area (Fukunaga et al., 1996). In addition, the vastus lateralis MT was a good predictor of the voluntary knee extensor torque in patients with SCP and in TD children (Moreau et al., 2010). Still other studies reported no strong relationship between muscle size (i.e., muscle volume and anatomical cross-sectional area of the knee flexors and extensors) and strength in children with SCP (Reid et al., 2015). Since children with SCP display reduced specific tension (torque/unit CSA) compared to their TD peers, the association between plantar flexor muscle size and force output during gait may critically depend on neural recruitment issues (Elder et al., 2003). Yet, this has not been evaluated in this study. The absent relations in the TD children might reflect the rather homogenous and unconstrained gait patterns but could also reveal a greater dependency on their tendons for locomotion by using the passive elastic energy storage and recovery to reduce muscle work.

Regarding the divergent findings on eccentric fascicle lengthening, our results suggest that a low isometric strength level (e.g., also due to recruitment issues) may be related to the extent of eccentric excursion experienced by the gastrocnemius fascicles. Therefore, our finding is in line with the hypothesis as already formulated by Barber et al. (2017). In the past, the idea was raised that an eccentric overloading of the muscles may be a primary mechanism for an altered adaptation in connective tissue properties in children with SCP (Gough and Shortland, 2012), eventually negatively influencing factors such as the joint range of motion. In general, eccentric training appears to be beneficial for growth of healthy muscle fiber length *via* sarcomerogenesis (Lynn and Morgan, 1994; Butterfield et al., 2005) but perturbs fiber mechanics inducing myofibrillar remodeling (Carlsson et al., 2007; Franchi et al., 2014). Upon chronic eccentric overloading, insufficient repair process and substitution of myofibers by nonfunctional fibrotic tissue may be promoted (Zhu et al., 2007; Serrano and Muñoz-Cánoves, 2010). Our current finding supports the idea that weaker children with SCP may experience greater eccentric fascicle lengthening during gait. However, whether this actually overloads their muscles remains to be investigated. Nevertheless, we suggest focusing on efficient strengthening strategies for individuals with SCP.

We also analyzed the relationship of the foot landing angle (the steeper the more forefoot landing) and the extent of eccentric fascicle excursion. The current analysis demonstrated no significant correlation. Our sample might include less severe toes walkers than those included in the study of Barber et al. (2017); however, our finding agrees with previous studies that assessed the effect of voluntary toe-walking on the muscle-tendon behavior in healthy populations in which the stretch of the plantar flexors was fully taken up by the tendon (Lorentzen et al., 2018). Nevertheless, we did not examine the Achilles tendon (AT) behavior. Interestingly, in habitual high heel wearers, a model for chronically induced calf muscle shortening and declines in fascicle length (Csapo et al., 2010), similar to SCP, larger dynamic fascicles strains have been reported when walking on their toes. This would be analogous to Barber et al. (2017). Corresponding to the current findings, in simulated toe walking, both the soleus and gastrocnemius muscles seem to operate on the ascending limb of their length tension curve (Neptune et al., 2007). Therefore, it could be decisive, where on their individual force-length relationship the fascicles need to operate, which is a potential research goal for a further study.

Noteworthily, the correlation between resting fascicle length and peak push-off power in SCP did not reach significance, which is similar to Martin Lorenzo et al. (2018). However, when relating fascicle length during gait to their resting length, children with SCP that operated at a shorter relative length were able to produce larger ankle power during push-off (Table 3). Based on findings of reduced sarcomeres in series in the lower limb muscles of individuals with SCP, e.g., for the gastrocnemius (Mathewson et al., 2014) and soleus (Mathewson et al., 2014; Mathewson and Lieber, 2015), it can be assumed that the sarcomeres may likely need to operate with little overlap in SCP. Thus, adapting a shorter relative fascicle length during gait, as shown in this study, could be a compensatory strategy to shift the sarcomere operating region on the individual optimal part of the length-tension relationship. Therefore, fascicle and sarcomere lengths should not be equated. To the best of our knowledge, there is no actual experimental data available showing on which part of the length-tension curve the fascicles operate in individuals with SCP during gait. However, experimental instrumented strength tests showed that the maximal plantar flexor force generating capacity of SCP patients is shifted toward plantar flexion angles (Brouwer et al., 1998; Barber et al., 2012; Frisk et al., 2019). Thus, the eccentric fascicle excursions seen in SCP patients most likely reflect an individually excessed demand on weak muscles in unfavorable conditions for active force production. Determining the fascicle force-length relationship experimentally simultaneous with ultrasound during gait, as already performed in healthy individuals (Rubenson et al., 2012; Bohm et al., 2018), could be a promising aim for future studies.

In TD children, a slower fascicle shortening velocity was correlated with a larger push-off power. The dependence of muscle force output on the velocity of the fibers has long been established. During ambulation, fascicles in the plantar flexors usually maintain a relatively low shortening velocity compared with that of the MTU, likely to operate in more favorable regions of the force-velocity curve where muscle efficiency seems to be highest (Lichtwark et al., 2007; Lichtwark and Wilson, 2008). In the past, it could be shown that, as TD children walk faster, ankle power output increases (Schwartz et al., 2008). Worth mentioning, this relationship can also be confirmed when additionally analyzed using the current cross-sectional data (*r* = 0.79, *p* < 0.001). Yet, during faster walking, the force generation ability of the plantar flexors usually decreases, coinciding with an increase in fiber shortening velocity and more activation to generate muscle force (Farris and Sawicki, 2012; Arnold et al., 2013). We suggest that, at the individually preferred walking speed, the behavior of muscles in healthy subjects is likely tuned to minimize consumption of metabolic energy for eliciting contractions. The ability to maintain slower contractile velocities for higher joint power production may thus be a key factor for the velocity healthy humans choose to walk at (Farris and Sawicki, 2012). Accordingly, an inverse relationship between higher SSW speed and lower GM fascicle shortening velocities has been previously also reported in young and old adults (Stenroth et al., 2017). Whether slower fascicle shortening speed may further indirectly reflect Achilles tendon compliance is still subject of scientific controversy (Lichtwark and Wilson, 2008; Arnold et al., 2013; Werkhausen et al., 2019). Interestingly, for children with SCP, there was no significant correlation between fascicle shortening velocity and push-off power in the present study (Figure 6B). Since adequate regulation by the central nervous system may be needed to control the operating length and velocity of muscles fascicles during gait by muscle activation (Bohm et al., 2018), we assume that these relationships might be disturbed by poor neural control in SCP patients. However, these aspects were outside the scope of the current analysis.

### Limitations

Some limitations need to be considered. First, the current sample is small but homogenous yet the analysis is limited by its cross-sectional design. The results should not be generalized to unilaterally affected children. We admit that solely on the basis of these observed cross-sectional associations in children with bilateral CP, we are unable to legitimately deduce a cause-and-effect relationship, but we provided biomechanical reasoning to explain our findings and also suggest future aspects that need to be explored. From this point of view, this study generates research goals. Analyzing prolonged walking in SCP to study the effect of fatigue and assessing tendon stiffness and fascicle force angle relationships in combination with ultrasound during gait could be aims for future studies.

Second, the pathology in SCP affects several muscles, both from a morphological and coordinative perspective. Thus, further interrelated factors (e.g., neural control) are likely to affect the currently investigated associations to gait kinematics and kinetics. We only focused on the GM muscle, yet other plantar flexors, e.g., the M. soleus, may also play an important role considering its larger volume (Noble et al., 2014) and the fact that the M. soleus contractile behavior was shown to be partly distinctive from the GM in healthy populations (Cronin et al., 2013). Simulations also pointed out that soleus contracture causes more plantar flexion (more equinus) in stance and also aggravates hip and knee flexion (Ong et al., 2019). Future studies may also shift their focus accordingly.

Third, it should be also noted that the gait and related muscle variables were assessed while the study participants walked at a self-selected speed over a level walkway (Kalsi et al., 2016; Barber et al., 2017) or barefoot at a self-selected speed on a treadmill (Hösl et al., 2016). Despite familiarization in both approaches (e.g., to become familiar with walking with the ultrasound probe attached to the leg), walking on a treadmill can affect the children’s gait. While no difference in GM muscle fascicle behavior (Cronin and Finni, 2013) as well as in EMG and kinematic patterns (Lorentzen et al., 2018) could be found between treadmill and overground gait in TD adults, some differences may exist for children with SCP: van der Krogt et al. (2015) reported that children with SCP might rely on their hip joint for generating power on treadmills, but how the treadmill may alter the push-off mechanism remains debatable. Albeit not being different between patients and controls, the alterations in walking speed could also have an impact on gait kinetics.

Fourth, the validity of handheld dynamometry for plantar flexor strength measurements may be debatable due to issue in selective motor control and due to the fact that the optimal point on the length-tension curve for active force generation might have potentially been missed in the children with SCP due to the chosen measurement position (ankle joint in neutral). However, despite that shortcoming, correlations could be found and we showed that muscle weakness might be a key factor for the GM muscle fascicle behavior.

Finally, we chose a resting position in which the knee and ankle joint angles were at 90° and assumed that the gastrocnemius fascicles were off-tension. We opted for this position, which allowed to standardize the angular alignment of the knee and ankle. It was chosen to offload the GM and to ensure that the fascicles were in slack. The gastrocnemius should be considerably off tension due to findings in TD subjects (De Monte et al., 2006) and in subjects with SCP (Park et al., 2019). Investigating healthy individuals, Hoffman et al. (2014) suggested that healthy GM fascicles operate at ~123–135% of their rest length during flat forward walking, which refers to the beginning of the descending limb of their active length-tension curve. In the present study, the GM fascicles of the TD children similarly operated on average at ~114–138% of their seated resting lengths, which supports the validity of our normalizing approach using the seated resting position for measuring resting fascicle length.

## Summary and Conclusion

Our results suggest that the gastrocnemius fascicle eccentric contractile behavior may be related to the extent of muscle weakness of patients with SCP. Since we did not find a relationship between the foot landing angle and the eccentric fascicle lengthening behavior, the results of Barber et al. (2017) and Hösl et al. (2016) may not contradict each other. In particular, children with SCP with weaker muscles may therefore be more susceptible to chronic eccentric overloading and pathological remodeling processes, which has to be clarified in future studies. Carefully speaking, the correlations also suggest that atrophy of gastrocnemius MT may be related to reduced plantar flexion and may promote undesired knee flexion during gait. Therefore, sole calculations of musculoskeletal models showing shorter gastrocnemius MTU lengths could be misleading in treatment decision-making. In addition, we provided evidence that a thicker gastrocnemius muscle belly and particularly longer gastrocnemius fascicles could be reasonable morphometric properties that should be targeted in interventions for pediatric and juvenile patients with SCP, since they may relate to a more upright and faster gait pattern. Despite longer fascicles are *per se* favorable for children with SCP, we found that those patients who managed to operate on shorter relative length with respect to their resting length produced a better push-off. The peak push-off power in individuals with SCP is likely affected by the fascicle force-length relationship (probably altered sarcomere length and number), while in TD individuals the force-velocity relationship could instead be decisive.

## Data Availability Statement

Data supporting the major conclusions of this manuscript can be made available by the authors, upon reasonable requests.

## Ethics Statement

The studies involving human participants and were reviewed and approved by ethics committee at Technical University Munich Rechts der Isar. Written informed consent to participate in this study was provided by the participants and their legal guardian/next of kin.

## Author Contributions

AK and MH developed the idea, planned and designed the data reanalysis, and prepared the current manuscript. MH, AA, HB, and AZ planned and conducted the initial experimental protocol. MH analyzed the data, and AZ assisted in data collection and analysis during her Master’s Degree at the TU Munich. All authors contributed to both the interpretation and discussion of the results and critically revised and edited the manuscript. All authors read and approved the submitted version.

### Conflict of Interest

The authors declare that the research was conducted in the absence of any commercial or financial relationships that could be construed as a potential conflict of interest.

## References

[ref1] Aggeloussis N. Giannakou E. Albracht K. Arampatzis A. (2010). Reproducibility of fascicle length and pennation angle of gastrocnemius medialis in human gait in vivo. Gait Posture 31, 73–77. 10.1016/j.gaitpost.2009.08.249, PMID: 19775893

[ref2] Altman D. G. (1990). “Practical statistics for medical research” in Practical statistics for medical research. New York: Chapman and Hall/CRC, 404.

[ref3] Armand S. Decoulon G. Bonnefoy-Mazure A. (2016). Gait analysis in children with cerebral palsy. EFORT Open Rev. 1, 448–460. 10.1302/2058-5241.1.000052 28698802 PMC5489760

[ref4] Arnold A. S. Anderson F. C. Pandy M. G. Delp S. L. (2005). Muscular contributions to hip and knee extension during the single limb stance phase of normal gait: a framework for investigating the causes of crouch gait. J. Biomech. 38, 2181–2189. 10.1016/j.jbiomech.2004.09.036, PMID: 16154404

[ref5] Arnold E. M. Hamner S. R. Seth A. Millard M. Delp S. L. (2013). How muscle fiber lengths and velocities affect muscle force generation as humans walk and run at different speeds. J. Exp. Biol. 216, 2150–2160. 10.1242/jeb.075697, PMID: 23470656 PMC3656509

[ref6] Barber L. Barrett R. Lichtwark G. (2012). Medial gastrocnemius muscle fascicle active torque-length and Achilles tendon properties in young adults with spastic cerebral palsy. J. Biomech. 45, 2526–2530. 10.1016/j.jbiomech.2012.07.018, PMID: 22867763

[ref7] Barber L. Carty C. Modenese L. Walsh J. Boyd R. Lichtwark G. (2017). Medial gastrocnemius and soleus muscle-tendon unit, fascicle, and tendon interaction during walking in children with cerebral palsy. Dev. Med. Child Neurol. 59, 843–851. 10.1111/dmcn.13427, PMID: 28369824

[ref8] Barber L. Hastings-Ison T. Baker R. Barrett R. Lichtwark G. (2011). Medial gastrocnemius muscle volume and fascicle length in children aged 2 to 5 years with cerebral palsy. Dev. Med. Child Neurol. 53, 543–548. 10.1111/j.1469-8749.2011.03913.x, PMID: 21506995

[ref9] Barber L. Hastings-Ison T. Baker R. Kerr Graham H. Barrett R. Lichtwark G. (2013). The effects of botulinum toxin injection frequency on calf muscle growth in young children with spastic cerebral palsy: a 12-month prospective study. J. Child. Orthop. 7, 425–433. 10.1007/s11832-013-0503-x, PMID: 24432106 PMC3838523

[ref10] Bell K. J. Ounpuu S. DeLuca P. A. Romness M. J. (2002). Natural progression of gait in children with cerebral palsy. J. Pediatr. Orthop. 22, 677–682. 10.1097/01241398-200209000-00020, PMID: 12198474

[ref11] Benard M. R. Becher J. G. Harlaar J. Huijing P. A. Jaspers R. T. (2009). Anatomical information is needed in ultrasound imaging of muscle to avoid potentially substantial errors in measurement of muscle geometry. Muscle Nerve 39, 652–665. 10.1002/mus.21287, PMID: 19291798

[ref12] Bland D. C. Prosser L. A. Bellini L. A. Alter K. E. Damiano D. L. (2011). Tibialis anterior architecture, strength, and gait in individuals with cerebral palsy. Muscle Nerve 44, 509–517. 10.1002/mus.22098, PMID: 21755515 PMC3175274

[ref13] Blazevich A. J. Sharp N. C. C. (2005). Understanding muscle architectural adaptation: macro‐ and micro-level research. Cells Tissues Organs 181, 1–10. 10.1159/000089964, PMID: 16439814

[ref14] Bohannon R. W. Smith M. B. (1987). Interrater reliability of a modified Ashworth scale of muscle spasticity. Phys. Ther. 67, 206–207. 10.1093/ptj/67.2.206 3809245

[ref15] Bohm S. Marzilger R. Mersmann F. Santuz A. Arampatzis A. (2018). Operating length and velocity of human vastus lateralis muscle during walking and running. Sci. Rep. 8:5066. 10.1038/s41598-018-23376-5, PMID: 29567999 PMC5864755

[ref16] Brouwer B. Wheeldon R. K. Stradiotto-Parker N. Allum J. (1998). Reflex excitability and isometric force production in cerebral palsy: the effect of serial casting. Dev. Med. Child Neurol. 40, 168–175. 10.1111/j.1469-8749.1998.tb15442.x, PMID: 9566653

[ref17] Butterfield T. A. (2010). Eccentric exercise in vivo: strain-induced muscle damage and adaptation in a stable system. Exerc. Sport Sci. Rev. 38, 51–60. 10.1097/JES.0b013e3181d496eb, PMID: 20335736

[ref18] Butterfield T. A. Leonard T. R. Herzog W. (2005). Differential serial sarcomere number adaptations in knee extensor muscles of rats is contraction type dependent. J. Appl. Physiol. 99, 1352–1358. 10.1152/japplphysiol.00481.2005, PMID: 15947030

[ref19] Carlsson L. Yu J. -G. Moza M. Carpen O. Thornell L. -E. (2007). Myotilin: a prominent marker of myofibrillar remodelling. Neuromuscul. Disord. 17, 61–68. 10.1016/j.nmd.2006.09.007, PMID: 17056257

[ref20] Choe Y. R. Kim J. S. Kim K. H. Yi T. I. (2018). Relationship between functional level and muscle thickness in young children with cerebral palsy. Ann. Rehabil. Med. 42, 286–295. 10.5535/arm.2018.42.2.286, PMID: 29765882 PMC5940605

[ref21] Cronin N. J. Avela J. Finni T. Peltonen J. (2013). Differences in contractile behaviour between the soleus and medial gastrocnemius muscles during human walking. J. Exp. Biol. 216, 909–914. 10.1242/jeb.078196, PMID: 23197091

[ref22] Cronin N. J. Finni T. (2013). Treadmill versus overground and barefoot versus shod comparisons of triceps surae fascicle behaviour in human walking and running. Gait Posture 38, 528–533. 10.1016/j.gaitpost.2013.01.027, PMID: 23473808

[ref23] Csapo R. Maganaris C. N. Seynnes O. R. Narici M. V. (2010). On muscle, tendon and high heels. J. Exp. Biol. 213, 2582–2588. 10.1242/jeb.044271, PMID: 20639419

[ref24] Dallmeijer A. J. Baker R. Dodd K. J. Taylor N. F. (2011). Association between isometric muscle strength and gait joint kinetics in adolescents and young adults with cerebral palsy. Gait Posture 33, 326–332. 10.1016/j.gaitpost.2010.10.092, PMID: 21185726

[ref25] Damiano D. L. Martellotta T. L. Sullivan D. J. Granata K. P. Abel M. F. (2000). Muscle force production and functional performance in spastic cerebral palsy: relationship of cocontraction. Arch. Phys. Med. Rehabil. 81, 895–900. 10.1053/apmr.2000.5579, PMID: 10896001

[ref27] Delp S. L. Arnold A. S. Speers R. A. Moore C. A. (1996). Hamstrings and psoas lengths during normal and crouch gait: implications for muscle-tendon surgery. J. Orthop. Res. 14, 144–151. 10.1002/jor.1100140123, PMID: 8618157

[ref26] De Monte G. Arampatzis A. Stogiannari C. Karamanidis K. (2006). In vivo motion transmission in the inactive gastrocnemius medialis muscle-tendon unit during ankle and knee joint rotation. J. Electromyogr. Kinesiol. 16, 413–422. 10.1016/j.jelekin.2005.10.001, PMID: 16309922

[ref28] Downing A. L. Ganley K. J. Fay D. R. Abbas J. J. (2009). Temporal characteristics of lower extremity moment generation in children with cerebral palsy. Muscle Nerve 39, 800–809. 10.1002/mus.21231, PMID: 19260049 PMC2771761

[ref30] Eames N. W. A. Baker R. J. Cosgrove A. P. (1997). Defining gastrocnemius length in ambulant children. Gait Posture 6, 9–17. 10.1016/S0966-6362(96)01105-8

[ref31] Eek M. N. Tranberg R. Beckung E. (2011). Muscle strength and kinetic gait pattern in children with bilateral spastic CP. Gait Posture 33, 333–337. 10.1016/j.gaitpost.2010.10.093, PMID: 21168334

[ref32] Elder G. C. B. Kirk J. Stewart G. Cook K. Weir D. Marshall A.. (2003). Contributing factors to muscle weakness in children with cerebral palsy. Dev. Med. Child Neurol. 45, 542–550. 10.1017/s0012162203000999, PMID: 12882533

[ref33] Farris D. J. Sawicki G. S. (2012). Human medial gastrocnemius force-velocity behavior shifts with locomotion speed and gait. Proc. Natl. Acad. Sci. U. S. A. 109, 977–982. 10.1073/pnas.1107972109, PMID: 22219360 PMC3271879

[ref34] Franchi M. V. Atherton P. J. Reeves N. D. Flück M. Williams J. Mitchell W. K.. (2014). Architectural, functional and molecular responses to concentric and eccentric loading in human skeletal muscle. Acta Physiol. 210, 642–654. 10.1111/apha.12225, PMID: 24387247

[ref35] Francis C. A. Lenz A. L. Lenhart R. L. Thelen D. G. (2013). The modulation of forward propulsion, vertical support, and center of pressure by the plantarflexors during human walking. Gait Posture 38, 993–997. 10.1016/j.gaitpost.2013.05.009, PMID: 23787149 PMC3795949

[ref36] Frisk R. F. Lorentzen J. Barber L. Nielsen J. (2019). Characterization of torque generating properties of ankle plantar flexor muscles in ambulant adults with cerebral palsy. Eur. J. Appl. Physiol. 119, 1127–1136. 10.1007/s00421-019-04102-z, PMID: 30778762

[ref38] Fukunaga T. Kubo K. Kawakami Y. Fukashiro S. Kanehisa H. Maganaris C. N. (2001). In vivo behaviour of human muscle tendon during walking. Proc. Biol. Sci. 268, 229–233. 10.1098/rspb.2000.1361, PMID: 11217891 PMC1088596

[ref39] Fukunaga T. Roy R. R. Shellock F. G. Hodgson J. A. Edgerton V. R. (1996). Specific tension of human plantar flexors and dorsiflexors. J. Appl. Physiol. 80, 158–165. 10.1152/jappl.1996.80.1.158, PMID: 8847297

[ref40] Gao F. Zhao H. Gaebler-Spira D. Zhang L. Q. (2011). In vivo evaluations of morphologic changes of gastrocnemius muscle fascicles and achilles tendon in children with cerebral palsy. Am. J. Phys. Med. Rehabil. 90, 364–371. 10.1097/PHM.0b013e318214f699, PMID: 21765255

[ref41] Gillett J. G. Barrett R. S. Lichtwark G. A. (2013). Reliability and accuracy of an automated tracking algorithm to measure controlled passive and active muscle fascicle length changes from ultrasound. Comput. Methods Biomech. Biomed. Engin. 16, 678–687. 10.1080/10255842.2011.633516, PMID: 22235878

[ref42] Gough M. Eve L. C. Robinson R. O. Shortland A. (2004). Short-term outcome of multilevel surgical intervention in spastic diplegic cerebral palsy compared with the natural history. Dev. Med. Child Neurol. 46, 91–97. 10.1017/s0012162204000192, PMID: 14974633

[ref43] Gough M. Shortland A. P. (2012). Could muscle deformity in children with spastic cerebral palsy be related to an impairment of muscle growth and altered adaptation? Dev. Med. Child Neurol. 54, 495–499. 10.1111/j.1469-8749.2012.04229.x, PMID: 22364585

[ref44] Graham H. K. Rosenbaum P. Paneth N. Dan B. Lin J. -P. Damiano D. L.. (2016). Cerebral palsy. Nat. Rev. Dis. Primers 2:15082. 10.1038/nrdp.2015.82, PMID: 27188686 PMC9619297

[ref45] Herskind A. Ritterband-Rosenbaum A. Willerslev-Olsen M. Lorentzen J. Hanson L. Lichtwark G.. (2016). Muscle growth is reduced in 15-month-old children with cerebral palsy. Dev. Med. Child Neurol. 58, 485–491. 10.1111/dmcn.12950, PMID: 26510820

[ref46] Hicks J. L. Schwartz M. H. Arnold A. S. Delp S. L. (2008). Crouched postures reduce the capacity of muscles to extend the hip and knee during the single-limb stance phase of gait. J. Biomech. 41, 960–967. 10.1016/j.jbiomech.2008.01.002, PMID: 18291404 PMC2443703

[ref47] Hoffman B. W. Cresswell A. G. Carroll T. J. Lichtwark G. A. (2014). Muscle fascicle strains in human gastrocnemius during backward downhill walking. J. Appl. Physiol. 116, 1455–1462. 10.1152/japplphysiol.01431.2012, PMID: 23558392

[ref48] Hösl M. Bohm H. Arampatzis A. Doderlein L. (2015). Effects of ankle-foot braces on medial gastrocnemius morphometrics and gait in children with cerebral palsy. J. Child. Orthop. 9, 209–219. 10.1007/s11832-015-0664-x, PMID: 26108740 PMC4486505

[ref49] Hösl M. Bohm H. Arampatzis A. Keymer A. Doderlein L. (2016). Contractile behavior of the medial gastrocnemius in children with bilateral spastic cerebral palsy during forward, uphill and backward-downhill gait. Clin. Biomech. 36, 32–39. 10.1016/j.clinbiomech.2016.05.008, PMID: 27208665

[ref50] Ishikawa M. Komi P. V. Grey M. J. Lepola V. Bruggemann G. -P. (2005). Muscle-tendon interaction and elastic energy usage in human walking. J. Appl. Physiol. 99, 603–608. 10.1152/japplphysiol.00189.2005, PMID: 15845776

[ref51] Kalsi G. Fry N. R. Shortland A. P. (2016). Gastrocnemius muscle-tendon interaction during walking in typically-developing adults and children, and in children with spastic cerebral palsy. J. Biomech. 49, 3194–3199. 10.1016/j.jbiomech.2016.07.038, PMID: 27545082

[ref52] Kerr C. Parkes J. Stevenson M. Cosgrove A. P. McDowell B. C. (2008). Energy efficiency in gait, activity, participation, and health status in children with cerebral palsy. Dev. Med. Child Neurol. 50, 204–210. 10.1111/j.1469-8749.2008.02030.x, PMID: 18215192

[ref53] Kruse A. Schranz C. Tilp M. Svehlik M. (2018). Muscle and tendon morphology alterations in children and adolescents with mild forms of spastic cerebral palsy. BMC Pediatr. 18:156. 10.1186/s12887-018-1129-4, PMID: 29743109 PMC5941654

[ref54] Lee W. -Y. Park G. -Y. Kwon D. R. (2014). Comparison of treatment effects between children with spastic cerebral palsy under and over five years after botulinum toxin type a injection. Ann. Rehabil. Med. 38, 200–208. 10.5535/arm.2014.38.2.200, PMID: 24855614 PMC4026606

[ref55] Lenhart R. L. Francis C. A. Lenz A. L. Thelen D. G. (2014). Empirical evaluation of gastrocnemius and soleus function during walking. J. Biomech. 47, 2969–2974. 10.1016/j.jbiomech.2014.07.007, PMID: 25107666 PMC4228932

[ref56] Lichtwark G. A. Bougoulias K. Wilson A. M. (2007). Muscle fascicle and series elastic element length changes along the length of the human gastrocnemius during walking and running. J. Biomech. 40, 157–164. 10.1016/j.jbiomech.2005.10.035, PMID: 16364330

[ref57] Lichtwark G. A. Wilson A. M. (2008). Optimal muscle fascicle length and tendon stiffness for maximising gastrocnemius efficiency during human walking and running. J. Theor. Biol. 252, 662–673. 10.1016/j.jtbi.2008.01.018, PMID: 18374362

[ref58] Lieber R. L. Friden J. (2002). Spasticity causes a fundamental rearrangement of muscle-joint interaction. Muscle Nerve 25, 265–270. 10.1002/mus.10036, PMID: 11870696

[ref59] Lieber R. L. Friden J. (2018). Muscle contracture and passive mechanics in cerebral palsy. J. Appl. Physiol. 126, 1492–1501. 10.1152/japplphysiol.00278.2018, PMID: 30571285 PMC6589815

[ref60] Lorentzen J. Willerslev-Olsen M. Huche Larsen H. Svane C. Forman C. Frisk R.. (2018). Feedforward neural control of toe walking in humans. J. Physiol. 596, 2159–2172. 10.1113/JP275539, PMID: 29572934 PMC5983220

[ref61] Lynn R. Morgan D. L. (1994). Decline running produces more sarcomeres in rat vastus intermedius muscle fibers than does incline running. J. Appl. Physiol. 77, 1439–1444. 10.1152/jappl.1994.77.3.1439, PMID: 7836150

[ref62] Maas J. C. Huijing P. A. Dallmeijer A. J. Harlaar J. Jaspers R. T. Becher J. G. (2015). Decrease in ankle-foot dorsiflexion range of motion is related to increased knee flexion during gait in children with spastic cerebral palsy. J. Electromyogr. Kinesiol. 25, 339–346. 10.1016/j.jelekin.2014.10.015, PMID: 25553965

[ref63] Malaiya R. McNee A. E. Fry N. R. Eve L. C. Gough M. Shortland A. P. (2007). The morphology of the medial gastrocnemius in typically developing children and children with spastic hemiplegic cerebral palsy. J. Electromyogr. Kinesiol. 17, 657–663. 10.1016/j.jelekin.2007.02.009, PMID: 17459729

[ref64] Martin Lorenzo T. Rocon E. Martinez Caballero I. Lerma Lara S. (2018). Medial gastrocnemius structure and gait kinetics in spastic cerebral palsy and typically developing children: a cross-sectional study. Medicine 97:e10776. 10.1097/MD.0000000000010776, PMID: 29794756 PMC6392514

[ref65] Mathewson M. A. Chambers H. G. Girard P. J. Tenenhaus M. Schwartz A. K. Lieber R. L. (2014). Stiff muscle fibers in calf muscles of patients with cerebral palsy lead to high passive muscle stiffness. J. Orthop. Res. 32, 1667–1674. 10.1002/jor.22719, PMID: 25138654

[ref66] Mathewson M. A. Lieber R. L. (2015). Pathophysiology of muscle contractures in cerebral palsy. Phys. Med. Rehabil. Clin. N. Am. 26, 57–67. 10.1016/j.pmr.2014.09.005, PMID: 25479779 PMC4258234

[ref67] Moreau N. G. Simpson K. N. Teefey S. A. Damiano D. L. (2010). Muscle architecture predicts maximum strength and is related to activity levels in cerebral palsy. Phys. Ther. 90, 1619–1630. 10.2522/ptj.20090377, PMID: 20847035 PMC2967708

[ref68] Neptune R. R. Burnfield J. M. Mulroy S. J. (2007). The neuromuscular demands of toe walking: a forward dynamics simulation analysis. J. Biomech. 40, 1293–1300. 10.1016/j.jbiomech.2006.05.022, PMID: 16842801

[ref69] Neptune R. R. Kautz S. A. Zajac F. E. (2001). Contributions of the individual ankle plantar flexors to support, forward progression and swing initiation during walking. J. Biomech. 34, 1387–1398. 10.1016/s0021-9290(01)00105-1, PMID: 11672713

[ref70] Noble J. J. Fry N. R. Lewis A. P. Keevil S. F. Gough M. Shortland A. P. (2014). Lower limb muscle volumes in bilateral spastic cerebral palsy. Brain and Development 36, 294–300. 10.1016/j.braindev.2013.05.008, PMID: 23790825

[ref71] O’Brien T. D. (2016). Musculoskeletal proportionality, biomechanical considerations, and their contribution to movement in adults and children. Pediatr. Exerc. Sci. 28, 210–216. 10.1123/pes.2015-0160, PMID: 27137167

[ref72] Ohata K. Tsuboyama T. Ichihashi N. Minami S. (2006). Measurement of muscle thickness as quantitative muscle evaluation for adults with severe cerebral palsy. Phys. Ther. 86, 1231–1239. 10.2522/ptj.20050189, PMID: 16959671

[ref73] Ong C. F. Geijtenbeek T. Hicks J. L. Delp S. L. (2019). Predicting gait adaptations due to ankle plantarflexor muscle weakness and contracture using physics-based musculoskeletal simulations. PLoS Comput. Biol. 15:e1006993. 10.1371/journal.pcbi.1006993, PMID: 31589597 PMC6797212

[ref74] Orendurff M. S. Aiona M. D. Dorociak R. D. Pierce R. A. (2002). Length and force of the gastrocnemius and soleus during gait following tendo Achilles lengthenings in children with equinus. Gait Posture 15, 130–135. 10.1016/S0966-6362(01)00154-0, PMID: 11869906

[ref75] Panizzolo F. A. Green D. J. Lloyd D. G. Maiorana A. J. Rubenson J. (2013). Soleus fascicle length changes are conserved between young and old adults at their preferred walking speed. Gait Posture 38, 764–769. 10.1016/j.gaitpost.2013.03.021, PMID: 23642629

[ref76] Park K. -B. Joo S. Y. Park H. Rhee I. Shin J. -K. Abdel-Baki S. W.. (2019). Architecture of the triceps surae muscles complex in patients with spastic hemiplegia: implication for the limited utility of the Silfverskiöld test. J. Clin. Med. 8:2096. 10.3390/jcm8122096, PMID: 31805732 PMC6947161

[ref77] Park E. S. Sim E. Rha D. -W. Jung S. (2014). Architectural changes of the gastrocnemius muscle after botulinum toxin type A injection in children with cerebral palsy. Yonsei Med. J. 55, 1406–1412. 10.3349/ymj.2014.55.5.1406, PMID: 25048504 PMC4108831

[ref78] Pitcher C. A. Elliott C. M. Panizzolo F. A. Valentine J. P. Stannage K. Reid S. L. (2015). Ultrasound characterization of medial gastrocnemius tissue composition in children with spastic cerebral palsy. Muscle Nerve 52, 397–403. 10.1002/mus.24549, PMID: 25556656

[ref79] Ponten E. Gantelius S. Lieber R. L. (2007). Intraoperative muscle measurements reveal a relationship between contracture formation and muscle remodeling. Muscle Nerve 36, 47–54. 10.1002/mus.20780, PMID: 17410593

[ref80] Reid S. L. Pitcher C. A. Williams S. A. Licari M. K. Valentine J. P. Shipman P. J.. (2015). Does muscle size matter? The relationship between muscle size and strength in children with cerebral palsy. Disabil. Rehabil. 37, 579–584. 10.3109/09638288.2014.935492, PMID: 24989066

[ref81] Rethlefsen S. A. Blumstein G. Kay R. M. Dorey F. Wren T. A. L. (2017). Prevalence of specific gait abnormalities in children with cerebral palsy revisited: influence of age, prior surgery, and gross motor function classification system level. Dev. Med. Child Neurol. 59, 79–88. 10.1111/dmcn.13205, PMID: 27421715

[ref82] Rha D. -W. Cahill-Rowley K. Young J. Torburn L. Stephenson K. Rose J. (2016). Biomechanical and clinical correlates of stance-phase knee flexion in persons with spastic cerebral palsy. PM R 8, 11–18. 10.1016/j.pmrj.2015.06.003, PMID: 26079863

[ref83] Rose G. E. Lightbody K. A. Ferguson R. G. Walsh J. C. Robb J. E. (2010). Natural history of flexed knee gait in diplegic cerebral palsy evaluated by gait analysis in children who have not had surgery. Gait Posture 31, 351–354. 10.1016/j.gaitpost.2009.12.006, PMID: 20116253

[ref84] Ross S. A. Engsberg J. R. (2002). Relation between spasticity and strength in individuals with spastic diplegic cerebral palsy. Dev. Med. Child Neurol. 44, 148–157. 10.1017/s0012162201001852, PMID: 12005315

[ref85] Rubenson J. Pires N. J. Loi H. O. Pinniger G. J. Shannon D. G. (2012). On the ascent: the soleus operating length is conserved to the ascending limb of the force-length curve across gait mechanics in humans. J. Exp. Biol. 215, 3539–3551. 10.1242/jeb.070466, PMID: 22771749

[ref86] Santuz A. Ekizos A. Janshen L. Baltzopoulos V. Arampatzis A. (2017). On the methodological implications of extracting muscle synergies from human locomotion. Int. J. Neural Syst. 27:1750007. 10.1142/S0129065717500071, PMID: 27873551

[ref87] Schwartz M. H. Rozumalski A. Trost J. P. (2008). The effect of walking speed on the gait of typically developing children. J. Biomech. 41, 1639–1650. 10.1016/j.jbiomech.2008.03.015, PMID: 18466909

[ref88] Sees J. P. Miller F. (2013). Overview of foot deformity management in children with cerebral palsy. J. Child. Orthop. 7, 373–377. 10.1007/s11832-013-0509-4, PMID: 24432097 PMC3838514

[ref89] Serrano A. L. Muñoz-Cánoves P. (2010). Regulation and dysregulation of fibrosis in skeletal muscle. Exp. Cell Res. 316, 3050–3058. 10.1016/j.yexcr.2010.05.035, PMID: 20570674

[ref90] Shortland A. P. Fry N. R. Eve L. C. Gough M. (2004). Changes to medial gastrocnemius architecture after surgical intervention in spastic diplegia. Dev. Med. Child Neurol. 46, 667–673. 10.1017/s0012162204001124, PMID: 15473170

[ref91] Šidák Z. K. (1967). Rectangular confidence regions for the means of multivariate normal distributions. J. Am. Stat. Assoc. 62, 626–633. 10.2307/2283989

[ref92] Smith L. R. Lee K. S. Ward S. R. Chambers H. G. Lieber R. L. (2011). Hamstring contractures in children with spastic cerebral palsy result from a stiffer extracellular matrix and increased in vivo sarcomere length. J. Physiol. 589, 2625–2639. 10.1113/jphysiol.2010.203364, PMID: 21486759 PMC3115830

[ref93] Stackhouse S. K. Binder-Macleod S. A. Lee S. C. K. (2005). Voluntary muscle activation, contractile properties, and fatigability in children with and without cerebral palsy. Muscle Nerve 31, 594–601. 10.1002/mus.20302, PMID: 15779003 PMC3069850

[ref94] Steele K. M. Seth A. Hicks J. L. Schwartz M. H. Delp S. L. (2013). Muscle contributions to vertical and fore-aft accelerations are altered in subjects with crouch gait. Gait Posture 38, 86–91. 10.1016/j.gaitpost.2012.10.019, PMID: 23200083 PMC3600387

[ref95] Stenroth L. Sipila S. Finni T. Cronin N. J. (2017). Slower walking speed in older men improves triceps surae force generation ability. Med. Sci. Sports Exerc. 49, 158–166. 10.1249/MSS.0000000000001065, PMID: 27471788

[ref96] Stief F. Bohm H. Michel K. Schwirtz A. Doderlein L. (2013). Reliability and accuracy in three-dimensional gait analysis: a comparison of two lower body protocols. J. Appl. Biomech. 29, 105–111. 10.1123/jab.29.1.105, PMID: 22813723

[ref97] van der Krogt M. Sloot L. H. Buizer A. I. Harlaar J. (2015). Kinetic comparison of walking on a treadmill versus over ground in children with cerebral palsy. J. Biomech. 48, 3577–3583. 10.1016/j.jbiomech.2015.07.046, PMID: 26315918

[ref98] Werkhausen A. Cronin N. J. Albracht K. Paulsen G. Larsen A. V. Bojsen-Møller J.. (2019). Training-induced increase in Achilles tendon stiffness affects tendon strain pattern during running. PeerJ 7:e6764. 10.7717/peerj.6764, PMID: 31086731 PMC6486809

[ref99] Wiley M. E. Damiano D. L. (1998). Lower-extremity strength profiles in spastic cerebral palsy. Dev. Med. Child Neurol. 40, 100–107. 10.1111/j.1469-8749.1998.tb15369.x, PMID: 9489498

[ref100] Wren T. A. L. Cheatwood A. P. Rethlefsen S. A. Hara R. Perez F. J. Kay R. M. (2010). Achilles tendon length and medial gastrocnemius architecture in children with cerebral palsy and equinus gait. J. Pediatr. Orthop. 30, 479–484. 10.1097/BPO.0b013e3181e00c80, PMID: 20574267

[ref101] Wren T. A. L. Do K. P. Kay R. M. (2004). Gastrocnemius and soleus lengths in cerebral palsy equinus gait--differences between children with and without static contracture and effects of gastrocnemius recession. J. Biomech. 37, 1321–1327. 10.1016/j.jbiomech.2003.12.035, PMID: 15275839

[ref102] Wren T. A. L. Rethlefsen S. Kay R. M. (2005). Prevalence of specific gait abnormalities in children with cerebral palsy: influence of cerebral palsy subtype, age, and previous surgery. J. Pediatr. Orthop. 25, 79–83. 10.1097/00004694-200501000-00018, PMID: 15614065

[ref103] Zhou J. Butler E. E. Rose J. (2017). Neurologic correlates of gait abnormalities in cerebral palsy: implications for treatment. Front. Hum. Neurosci. 11:103. 10.3389/fnhum.2017.00103, PMID: 28367118 PMC5355477

[ref104] Zhu J. Li Y. Shen W. Qiao C. Ambrosio F. Lavasani M.. (2007). Relationships between transforming growth factor-beta1, myostatin, and decorin: implications for skeletal muscle fibrosis. J. Biol. Chem. 282, 25852–25863. 10.1074/jbc.M704146200, PMID: 17597062

